# Cultures for acid-fast bacilli: are we being good stewards?

**DOI:** 10.1017/ash.2024.463

**Published:** 2024-12-30

**Authors:** Jennifer Hanrahan, Julie D. Sill, Patricia Ver Schneider, Jessica F. Copeland, Elsie Frimpongmaa Amoako-Kissi, Ogochukwu O. Ilobi, Angela J. Toepp

**Affiliations:** 1 Macon and Joan Brock Virginia Health Sciences, Eastern Virginia Medical School at Old Dominion University, Norfolk, VA, USA; 2 Sentara Health, Enterprise Analytics, Virginia Beach, VA, USA

## Abstract

**Objective:**

To describe the use of cultures for acid-fast bacilli (AFB) and situations in which AFB cultures are unlikely to be of clinical benefit.

**Design:**

Retrospective descriptive study of AFB cultures submitted to Sentara Health microbiology laboratory from December 1, 2021, to December 1, 2023. Data were collected from the electronic medical record and included patient demographics, the service line that ordered the culture, specimen source, and culture results.

**Setting:**

Sentara Healthcare System.

**Patients:**

All patients who had specimens submitted to the microbiology laboratory during the study period were included.

**Results:**

A total of 13,944 AFB cultures from 8,243 patients were collected during the study period. Of these, 4.72% (n = 389) patients had a positive result, and 40 of 680 positive cultures were likely contaminants or non-mycobacterial. The average number of days between culture collection and positive results was 84.32 days (SD = 49.64) and 56.25 days (SD = 8.32) for negative results. Most cultures were ordered by medical subspecialties (44.06%, n = 6,144), followed by orthopedic providers (23.34%, n = 3,254) and surgical subspecialty providers (16.11%, n = 2,246). Most specimens were pulmonary (n = 6,620) with 619 (9.35%) positive cultures. Of 3,561 AFB cultures ordered from bone specimens, only 17 were positive (0.48%). The number of specimens processed by the microbiology laboratory required 2 full-time microbiology technicians to process specimens.

**Conclusions:**

Many AFB cultures were sent from patients who did not have clinical scenarios consistent with mycobacterial disease and cultures were not clinically indicated. Implementation of testing criteria could decrease AFB cultures and healthcare costs.

## Introduction

Although mycobacterial infections are extremely important to diagnose, cultures for acid-fast bacilli (AFB) are sometimes ordered on specimens where they are not indicated, and obtaining a positive result would not change management. Mycobacteria are unusual pathogens and account for a low percentage of infection in humans. Cultures for AFB are often sent from surgical specimens in situations where the prior probability of infection is low.^1–12^ Submitting these cultures routinely is not cost effective and takes resources in terms of time for microbiology laboratory personnel as well as adding unnecessary costs for patients. Most clinical microbiology laboratories are not able to perform identification and sensitivity testing and must send these cultures out to reference laboratories. Furthermore, not all mycobacteria are clinically relevant, and sending out all positive cultures for identification and sensitivity is not cost effective. Nontuberculous mycobacteria (NTM) are most often isolated from sputum cultures, and patients may not necessarily have pulmonary disease due to mycobacteria. One respiratory specimen yielding NTM is not sufficient to establish the diagnosis of disease due to NTM,^13^ and obtaining sensitivities may not be clinically necessary. The long delay between identification by the outside laboratory and the appearance of the laboratory results in the medical record leads to further delay. Characterization of the types of specimens likely to lead to clinically actionable results could help improve cost-effective utilization of this laboratory test. We sought to describe the overall utilization of AFB cultures in our healthcare system in order to identify situations in which the tests may be overutilized.

## Methods

### Study design

We conducted a retrospective descriptive study comparing the incidence of positive AFB results to the incidence of negative AFB results as categorized by patient demographics, service line providers, and specimen sources. We also examined and described the species of organisms found in positive cultures. Additionally, we measured the time in days between culture collection and the result. Institutional review board approval was obtained prior to collecting any data.

### Setting

This study was conducted as a partnership between Eastern Virginia Medical School and Sentara Health, a not-for-profit healthcare organization based in Norfolk, Virginia. Sentara Health offers services in 12 acute care hospitals, 10 nursing centers, and 3 assisted living facilities serving Virginia and northeastern North Carolina.

### Patients

The study included all patients within the Sentara Health system who had specimens sent for AFB cultures between December 1, 2021, and December 1, 2023. AFB cultures ordered but not resulted (eg, no specimen was sent to the microbiology lab, and no result was available) were excluded.

### Data

Data were collected from the health system electronic medical record (EMR) and included patient age, sex at birth, race, ethnicity, service line that ordered the culture, specimen source, AFB lab date/times collected and resulted, and species of positive cultures. Service lines and species were grouped for ease of analysis. Data were stored securely in the Research Data Capture Tool.

### Statistical analysis

Patient-level data were described both in aggregate and stratified between patients with at least 1 positive culture and patients with no positive culture. Culture-level data were described for provider specialty groups and specimen sources both in aggregate and stratified between positive, negative, and nonpathogenic/contaminant results. Turnaround times between culture collection and result for positive cultures were described by species and were analyzed between positive and negative results. All data were analyzed using RStudio version 4.1.0. tableone package for descriptive statistics and ggplot2 package for figures.

## Results

### Patients

During the study period, there were more than 18 million encounters and 350,000 inpatient admissions within the health system. AFB cultures were collected from both inpatient and outpatient encounters.

There were 13,944 total AFB cultures from 8,243 unique patients with results during the study period (December 1, 2021–December 1, 2023). Of these patients, (4.7%, n = 389) had at least one positive result, and the remaining patients (95.3%, n = 7,854) had negative results. Demographics of the patients are in Table 1. The average number of cultures collected per patient in the total AFB culture population was 1.69 (SD = 1.32); for patients with at least one positive culture, the average number of cultures collected per patient was 3.14 (SD = 2.15), and for patients with no positive cultures, the average number of cultures per patient was 1.62 (SD = 1.22).

**Table 1.** Overall patient demographics

Variable	n (%)
**N**	8,243
Sex at birth	
Female	3,909 (47.4)
Male	4,315 (52.3)
Unknown	19 (0.2)
Race	
American Indian	18 (0.2)
Asian	241 (2.9)
Black	2,241 (27.2)
Other unknown	45 (0.5)
Pacific Islander	43 (0.5)
White	5,655 (68.6)
Ethnicity	
Hispanic or Latino	315 (3.8)
Not Hispanic or Latino	7891 (95.7)
Unknown	37 (0.4)
History of lung disease	3,115 (37.8)
Patients with positive cultures	389 (4.7)
Patients with negative cultures	8,061 (97.8)
Patients with other/non-pathogen cultures^*^	40 (0.5)
Patients with at least 2 cultures all negative	2,545 (30.9)
**Variable**	**Mean (SD)**
Average age per patient	62.52 (15.96)
Average # admissions per patient	1.18 (0.60)
Average # cultures per patient	1.69 (1.32)
Average # days culture collected to result	57.68 (15.08)
Average # days culture collected to positive result	84.32 (49.64)
Average # days culture collected to negative result	56.25 (8.32)

* *Streptomyces* species, partially acid-fast bacillus, *Nocardia* species, *Mycobacterium gordonae*.

**Figure 1.**
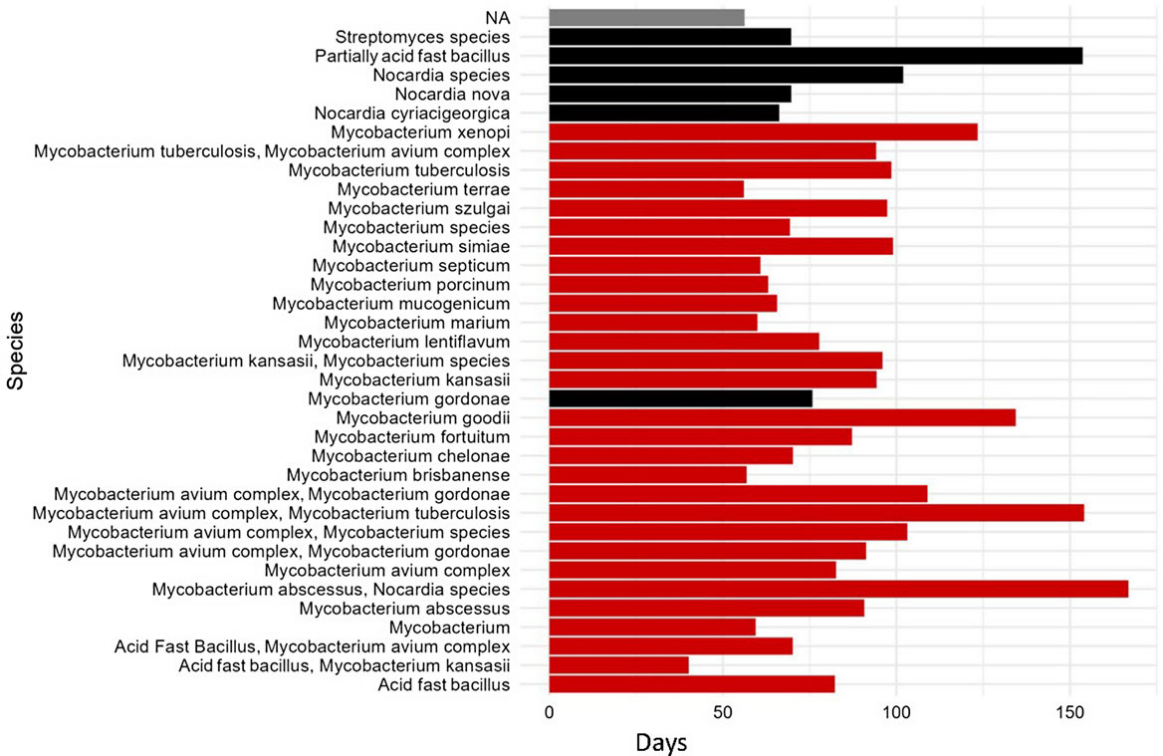
Acid-fast bacilli species by average number of days to identification.

The average number of days between culture collection and positive results was 84.32 days (SD = 49.64) and for negative results was 56.25 days (SD = 8.32). See Figure 1.

### Cultures

There were 13,944 total AFB cultures with results during the study period (December 1, 2021–December 1, 2023). Of those, 13,224 had negative results, 680 had positive results, and 40 had non-AFB, nonpathogenic, or contaminant results (*Streptomyces species, partially acid-fast bacillus, Nocardia species, Mycobacterium gordonae).

**Figure 2.**
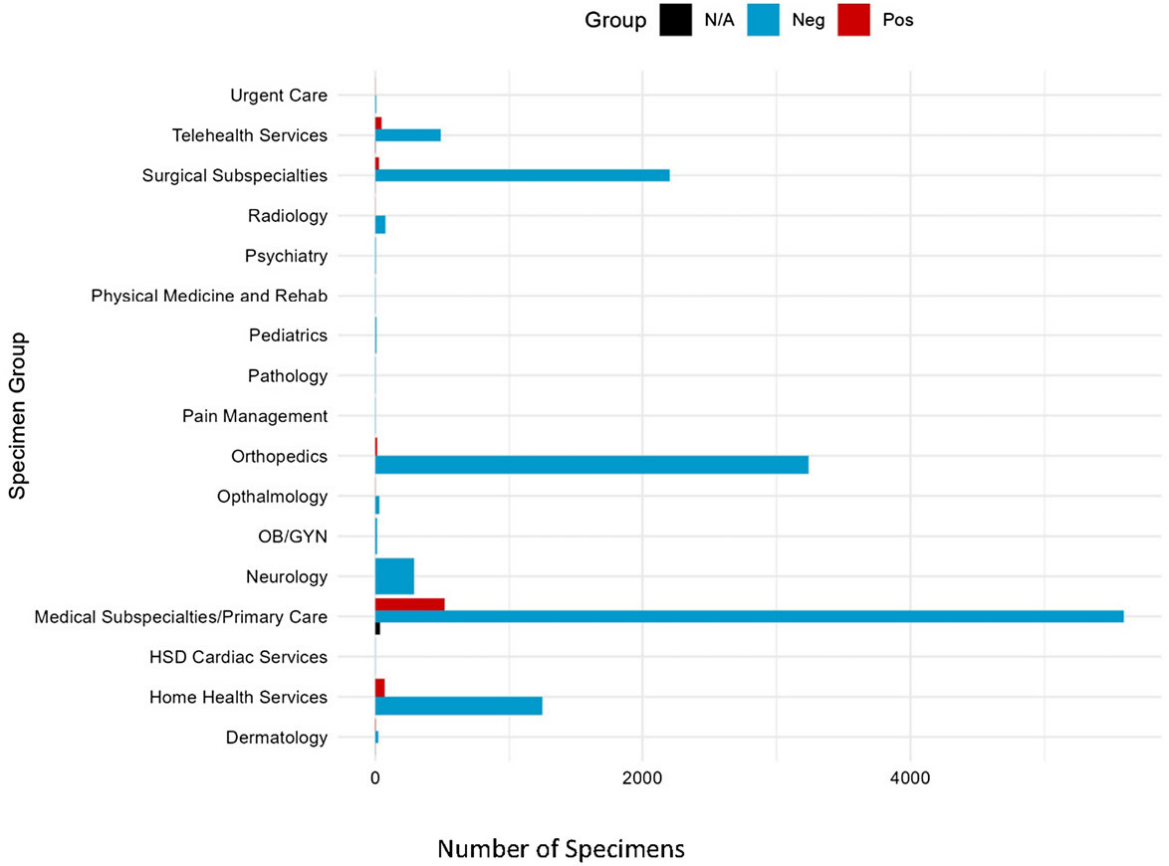
Acid-fast bacilli specimens sent by specialty group. N/A group is a contaminant or non-mycobacterial organism.

**Figure 3.**
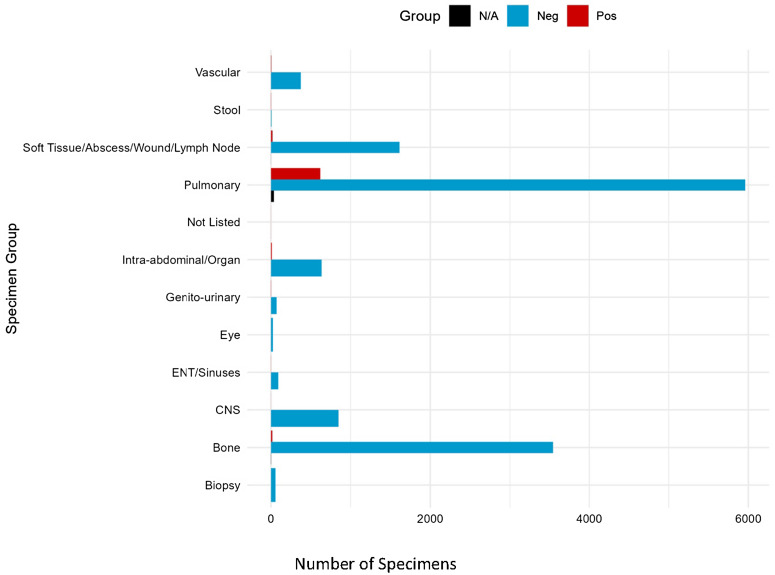
Number of specimens by specimen source. N/A are specimens that were either contaminants or non-mycobacterial organisms.

Most cultures were ordered by medical subspecialties/primary care providers (44.06%, n = 6,144), followed by orthopedic providers (23.34%, n = 3,254) and surgical subspecialty providers (16.11%, n = 2,246). Of those cultures with positive results, 75.88% (n = 516) were ordered by medical subspecialties/primary care providers (see Figure 2.) Most of the specimens collected came from pulmonary sources (47.45%, n = 6,617), followed by bone (25.54%, n = 3,561) and soft tissue/abscess/wound/lymph node sources (11.72%, n = 1,634). Of the cultures, the following were positive by source: pulmonary 619 of 5962 (10.38%), genito-urinary 3 of 71 (4.23%), intra-abdominal 10 of 636 (1.57%), soft tissue/abscess 19 of 1614 (1.18%), bone/synovial fluid 17 of 3561 (0.48%), and cerebrospinal fluid (CSF) 1 of 848 (0.11%) (see Figure 3 and Table 2).

**Table 2.** Culture by specialty and specimen groups by result

	n (%) Negative		n (%) Positive		n (%) Nonpathogenic/contaminant	
Variable	Specimens	Patients	Specimens	Patients	Specimens	Patients
N	13,224	8,631	680	413	40	41
Specialty group (%)						
Dermatology	22 (0.2)	21 (0.2)	3 (0.4)	2 (0.5)	1 (2.5)	1 (2.4)
Medical subspecialties/primary care	5,592 (42.3)	3,624 (42.0)	516 (75.9)	286 (69.2)	36 (90.0)	37 (90.2)
Neurology	289 (2.2)	280 (3.2)	0 (0.0)	0 (0.0)	0 (0.0)	0 (0.0)
Ophthalmology	29 (0.2)	27 (0.3)	1 (0.1)	1 (0.2)	0 (0.0)	0 (0.0)
Orthopedics	3,240 (24.5)	1,699 (19.7)	14 (2.1)	14 (3.4)	0 (0.0)	0 (0.0)
Other	512 (3.9)	370 (4.3)	48 (7.1)	37 (9.0)	2 (5.0)	2 (4.9)
Outpatient	1,247 (9.4)	1,047 (12.1)	70 (10.3)	50 (12.1)	0 (0.0)	0 (0.0)
Radiology	75 (0.6)	72 (0.8)	1 (0.1)	1 (0.2)	0 (0.0)	0 (0.0)
Surgical subspecialties	2,218 (16.8)	1,491 (17.3)	27 (4.0)	22 (5.3)	1 (2.5)	1 (2.4)
	Specimens	Patients	Specimens	Patients	Specimens	Patients
N	13,224	8,721	680	402	40	40
Specimen group (%)						
Biopsy	57 (0.4)	39 (0.4)	0 (0.0)	0 (0.0)	0 (0.0)	0 (0.0)
Bone	3,541 (26.8)	2,003 (23.0)	17 (2.5)	17 (4.2)	3 (7.5)	2 (5.0)
Central Nervous System	848 (6.4)	785 (9.00)	1 (0.1)	1 (0.2)	0 (0.0)	0 (0.0)
Ear Nose and Throat/sinuses	91 (0.7)	76 (0.9)	2 (0.3)	2 (0.5)	0 (0.0)	0 (0.0)
Eye	23 (0.2)	23 (0.3)	0 (0.0)	0 (0.0)	0 (0.0)	0 (0.0)
Genito-urinary	71 (0.5)	59 (0.7)	3 (0.4)	3 (0.7)	0 (0.0)	0 (0.0)
Intra-abdominal/organ	636 (4.8)	558 (6.4)	10 (1.5)	9 (2.2)	0 (0.0)	0 (0.0)
Not listed	0 (0.0)	0 (0.0)	1 (0.1)	1 (0.2)	0 (0.0)	0 (0.0)
Pulmonary	5,962 (45.1)	3,696 (42.4)	619 (91.0)	345 (85.8)	36 (90.0)	37 (92.5)
Soft tissue/abscess/wound/lymph Node	1,614 (12.2)	1,176 (13.5)	19 (2.8)	18 (4.5)	1 (2.5)	1 (2.5)
Stool	7 (0.1)	7 (0.1)	3 (0.4)	3 (0.7)	0 (0.0)	0 (0.0)
Vascular	374 (2.8)	299 (3.4)	5 (0.7)	3 (0.7)	0 (0.0)	0 (0.0)

Only 0.48% of bone/synovial fluid cultures yielded a positive result. Of those, 5 of 17 were from lower extremities, and only 2 of the 5 were definite pathogens: one from a patient with chronic tuberculous arthritis of the ankle and one that grew *M. abscessus* from a chronic prosthetic knee infection. Two of the 5 cultures were from gangrenous toes that were amputated and grew *M. avium-intracellulare* (MAC) along with other bacteria, and one was from a prosthetic joint that grew coagulase-negative staphylococcus, *E.coli*, and MAC. MAC was suspected to be a contaminant but was treated for a year.

Among the CSF cultures, the one patient who had a positive culture had Pott’s disease and also had a paraspinal abscess that was culture positive for tuberculosis. Overall, the yield from non-respiratory cultures was low, and AFB cultures would not be considered clinically indicated in some of these patients.

## Conclusion

Ordering AFB cultures on specimens from patients who don’t have syndromes consistent with mycobacterial infection may lead to overutilization of AFB cultures. The microbiology laboratory in our system spends about 140 h per week on work dedicated to AFB cultures. If this work were decreased by 50% by setting constraints on ordering tests, the saving would be approximately $116,000 in hourly staff costs alone. These savings don’t include costs for laboratory space, supplies, utilities, or opportunity costs by not doing other tests that could be more beneficial.

Our findings suggest that there could be cost benefit in limiting testing for AFB cultures to reduce inefficient and ineffective use of laboratory resources, as well as opportunity benefit in being able to provide other tests that may be more clinically relevant in our setting. Providing education to medical practitioners about the role of these cultures, especially guidelines on clinical scenarios in which they are not warranted, may help reduce unnecessary time and spending. Other methods to limit testing include automatic blocking in the EMR,^15^ eliminating panels of tests, or making ordering more difficult.^16^

A strength of this study lies in the fact that all data analyzed came from real-world clinical settings within the recent past, which provides a substantial sample size with a good distribution across genders and ethnicities. Therefore, the findings of this study provide useful insight into where interventions may be helpful.

There are some limitations to this study. The appropriateness of the cultures ordered was not assessed on all patients. AFB cultures take weeks to provide a definitive result and require clinical correlation as NTMs are common environmental organisms. Follow-up is not available for all of the patients for whom cultures were sent, making it difficult to determine the percentage of tests that were true positive results versus contaminants or colonization.

As expected, most specimens in this study were from pulmonary sources. As with any culture result, clinical correlation is needed to differentiate colonization from infection. Although there has been an increase in infection due to NTMs,^18^ this would not account for all positive results obtained. It is likely that at least half of the NTMs obtained from pulmonary specimens represented colonization or contamination versus being true pathogens. Our results likely underestimate the magnitude of the positive culture results that were not pathogenic. In addition to excess costs and laboratory resources being used to identify bacteria that may not be clinically relevant, there are potential patient costs in terms of unnecessary exposure to antimicrobial therapy when it is difficult to decide whether an organism may be a pathogen and providers opt to treat it out of caution.

Implementing laboratory criteria for accepting specimens for AFB cultures and reviewing AFB cultures prior to having them undergo further testing for identification and sensitivity could reduce costs for the microbiology laboratory and liberate personnel to perform other clinical tests.
